# Dual Nitrergic/Cholinergic Control of Short-Term Plasticity of Corticostriatal Inputs to Striatal Projection Neurons

**DOI:** 10.3389/fncel.2015.00453

**Published:** 2015-11-23

**Authors:** Craig P. Blomeley, Sarah Cains, Enrico Bracci

**Affiliations:** ^1^Mill Hill Laboratory, The Francis Crick InstituteThe Ridgeway, London, UK; ^2^Department of Psychology, The University of SheffieldSheffield, UK

**Keywords:** striatum, cholinergic interneuron, nitrergic interneuron, nitric oxide, microcircuit

## Abstract

The ability of nitric oxide and acetylcholine to modulate the short-term plasticity of corticostriatal inputs was investigated using current-clamp recordings in BAC mouse brain slices. Glutamatergic responses were evoked by stimulation of corpus callosum in D1 and D2 dopamine receptor-expressing medium spiny neurons (D1-MSNs and D2-MSN, respectively). Paired-pulse stimulation (50 ms intervals) evoked depressing or facilitating responses in subgroups of both D1-MSNs and D2 MSNs. In both neuronal types, glutamatergic responses of cells that displayed paired-pulse depression were not significantly affected by the nitric oxide donor S-nitroso-N-acetylpenicillamine (SNAP; 100 μM). Conversely, in D1-MSNs and D2-MSNs that displayed paired-pulse facilitation, SNAP did not affect the first evoked response, but significantly reduced the amplitude of the second evoked EPSP, converting paired-pulse facilitation into paired-pulse depression. SNAP also strongly excited cholinergic interneurons and increased their cortical glutamatergic responses acting through a presynaptic mechanism. The effects of SNAP on glutamatergic response of D1-MSNs and D2-MSN were mediated by acetylcholine. The broad-spectrum muscarinic receptor antagonist atropine (25 μM) did not affect paired-pulse ratios and did not prevent the effects of SNAP. Conversely, the broad-spectrum nicotinic receptor antagonist tubocurarine (10 μM) fully mimicked and occluded the effects of SNAP. We concluded that phasic acetylcholine release mediates feedforward facilitation in MSNs through activation of nicotinic receptors on glutamatergic terminals and that nitric oxide, while increasing cholinergic interneurons’ firing, functionally impairs their ability to modulate glutamatergic inputs of MSNs. These results show that nitrergic and cholinergic transmission control the short-term plasticity of glutamatergic inputs in the striatum and reveal a novel cellular mechanism underlying paired-pulse facilitation in this area.

## Introduction

The striatum is crucially involved in motor control, action selection and reward-mediated learning (Graybiel, 2005). Striatal projection neurons are medium sized spiny neurons (MSNs) and belong to two distinct groups, referred to as direct and indirect pathway neurons, respectively. Direct pathway neurons project to basal ganglia output nuclei and express D1 dopamine receptors. Indirect pathway neurons project to intermediate basal ganglia nuclei and express D2 receptors (Kravitz et al., 2010). The activation of striatal projection neurons is determined by cortical and thalamic excitatory inputs, but local interneurons, that also receive cortical and thalamic excitatory inputs, play a crucial role in shaping the striatal output (Tepper and Bolam, 2004). While feed-forward inhibition provided by GABAergic interneurons in response to cortical inputs is relatively well understood (Wilson, 2007), the role of other interneurons is less understood. Cholinergic interneurons exert a complex influence in the striatal microcircuits, through activation of nicotinic and muscarinic receptors located both pre- and postsynaptically on several neuronal targets (Pakhotin and Bracci, 2007; Ding et al., 2010). Another striatal interneuronal type, characterized by the ability to generate low-threshold calcium spikes, is the only source of nitric oxide in the striatum and is also involved in information processing (Kawaguchi, 1993; Tepper and Bolam, 2004). Low-threshold calcium spike interneurons (LTSIs) are GABAergic and mediate feed-forward inhibition (Tepper and Bolam, 2004). However, they could also affect the striatal network through activation of nitric oxide signaling cascades. Interestingly, nitric oxide donors excite cholinergic interneurons (Centonze et al., 2001), suggesting that an interaction between nitric oxide and acetylcholine may play an important role in the local networks. To cast light on the ability of nitric oxide to modulate the striatal microcircuits, we stimulated glutamatergic fibers while recording from D1-MSNs, D2-MSNs and cholinergic interneurons before and after activation of nitric oxide signaling. We recorded MSNs in current-clamp conditions (with no current injected). This technique has the advantage of avoiding the well-known signal distortions arising from poor space-clamp of distal dendritic synaptic inputs (Spruston et al., 1993; Williams and Mitchell, 2008) and of preserving the dynamic interplay between synaptic inputs and dendritic voltage-dependent conductances, which may affect the transmembrane signal eventually reaching the perisomatic spike-generating region of the neuron.

## Materials and Methods

### Animals

All experiments were carried out in accordance with the UK Animals Act 1986 and were approved by the Animal Welfare Ethical Review Body of the University of Manchester (where the authors were previously employed) and the Ethical Review Committees of the University of Sheffield. Recordings were obtained from 28–63 day old male and female bacterial artificial chromosome (BAC) mice from either B6.Cg-Tg(Drd1a-tdTomato)6Calak/J (stock no. 016204; The Jackson Laboratory) or Drd2-eGFP (stock no. 000230-UNC; MMRRC) strains.

### Slice Preparation

Mice underwent cervical dislocation. Following rapid brain removal, parasagittal slices (250–300 μm-thick) were cut in a solution of artificial cerebrospinal fluid (ACSF) containing (in mM): 124 NaCl, 2 KCl, 1 MgSO4, 1.25 KH2PO4, 2 CaCl2, 26 NaHCO3, and 10 glucose), pH 7.2–7.4. Slices were oxygenated with 95% O_2_, 5% CO_2_ gas and maintained at 35°C for 30 min and subsequently at room temperature until used for recording.

### Electrophysiological Recordings

Individual slices were transferred to a submerged recording chamber and continuously superfused (2–3 ml/min at 25°C). Neurons expressing td-tomato fluorescent protein (TFP) or enhanced green fluorescent protein (eGFP) were identified through epifluorescent excitation with standard tomato/mCherry or GFP filters. Medium-sized neurons were identified as MSNs based on their distinctive electrophysiological properties (Gertler et al., 2008). In Drd1a-tdTomato mice, MSNs were identified as expressing D1-like or D2-like dopamine receptors based on the expression of TFP (D1-MSNs) or absence of it (D2-MSNs). In Drd2-eGFP mice, MSNs were classified as D1- or D2- MSNs based on the expression of eGFP (D2-MSNs) or absence of it (D1-MSNs).

Cholinergic interneurons were identified based on their large somata and their distinctive electrophysiological properties (Tepper and Bolam, 2004).

Experiments were carried out on D1-MSNs, D2-MSNs and cholinergic interneurons from both BAC strains, as specified in the Results. Data obtained from D1-MSNs, D2-MSNs and cholinergic interneurons in the two strains were pooled, as all the features tested in the study were similar in the two groups.

Whole-cell current-clamp (no current injected) and voltage-clamp recordings from dorsolateral striatal neurons were performed using glass pipettes containing (in mM): 120 K-gluconate, 20 KCl, 0.04 EGTA, 12 HEPES, 2 MgCl_2_, 4 Na2ATP, 0.4 NaGTP and 2 mg ml^−1^ neurobiotin (pH 7.3–7.4; osmolarity, 290–300 mosmol l^−1^). Pipette resistance was 3–5 MΩ. Whole-cell recordings were carried out either using bridge amplifiers (BA-1S, BA-01; NPI) connected to a micro1401 A/D converter or using an EPC10 HEKA amplifier and Patchmaster software (HEKA Electronik).

Cell-attached recordings were carried out from cholinergic interneurons identified by their large somata and the presence of spontaneous spikes. Spikes were detected as rapid biphasic deflections of the potential recorded with a bridge amplifier.

Miniature (mini) spontaneous EPSCs (mEPSCs) were recorded under voltage-clamp conditions (Vh was −80 mV for MSNs and −70 mV for cholinergic interneurons) and were defined as downward deflections of more than 5 pA from baseline. The analysis of spontaneous mEPSCs was performed using Minianalysis software (Synaptosoft). For each cell, at least 300 consecutive mEPSCs were considered for cumulative probability plots.

Experiments were performed in the continuous presence of a GABA_A_-receptor antagonist (either 10 μM gabazine or 50 μM picrotoxin) and a GABA_B_-receptor antagonist CGP52432 (2 μM). The ACSF containing these antagonists is referred to as control solution. For miniature EPSCs experiments, tetrodotoxin (1 μM) was also added. Drugs were obtained from Tocris or Sigma-Aldrich.

### Stimulation and Recording Protocols

Electrical stimulation was delivered using a mono-polar, ACSF-filled, glass electrode. Corticostriatal afferents were stimulated by placing the stimulation electrode in the corpus callosum (CC) as in previous studies (Logie et al., 2013). While this procedure is not completely selective for corticostriatal vs. thalamostriatal fibers, most activated axons can be assumed to be corticostriatal.

Stimulation intensity (200–500 μA, 0.1 ms) was set to evoke responses with amplitudes <10 mV in current-clamp experiments and >500 pA in voltage-clamp experiments.

For paired-pulse experiments, corticostriatal fibers were activated by two stimuli separated by 50 ms. Such paired-pulse stimulation was applied every 10 s without interruptions throughout the experiment. Evoked responses were recorded in whole-cell current-clamp mode as excitatory postsynaptic potentials (EPSPs) or in voltage-clamp mode as excitatory postsynaptic currents (EPSCs). During drug applications, delivery of cortical stimuli was continued but data acquired during the first 5 min of each application (wash-in period) were discarded.

Input resistance was constantly monitored in current-clamp or voltage clamp experiments by applying 300–500 ms hyperpolarizing current or voltage steps to the patched neuron. If the input resistance changed more than 20% the recording was discarded.

### Intracellular Staining with Neurobiotin

Recording electrodes were filled with a solution of 2% neurobiotin (Vector Laboratories). After the experiment, the slice was placed in 4% paraformaldehyde overnight. Neurons were stained using the VectaStain ABC Elite kit (Vector Laboratories) and 3,3-diaminobenzidine (DAB; Sigma). Slices were mounted on slides with Vectashield (Vector Laboratories), coverslipped and processed for microscopy.

### Data Analysis and Statistical Methods

Data were analyzed with Signal 2.16 (C.E.D.), Igor Pro 5.0 (WaveMetrics) and GraphPad Prism 6. Summary data are reported as mean ± SEM. Probability levels are indicated as (*p < 0.05, **p < 0.01, ***p < 0.001).

For individual neurons, the paired pulse ratio ([2nd response amplitude]:[1st response amplitude]) was calculated for each paired-pulse stimulation and averaged over all the consecutive trials that took place in a given pharmacological condition.

To determine the effects of a pharmacological treatment on evoked responses in each individual neuron, the first response amplitudes and the paired-pulse ratios measured in that cell in two different pharmacological conditions were compared using the Mann–Whitney U test.

## Results

### Effects of SNAP on D1-MSNs

**Figure 1:**
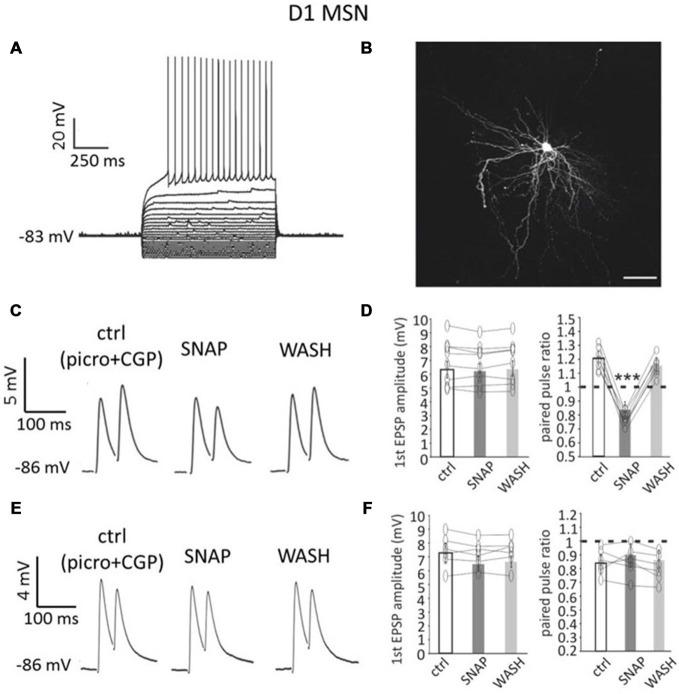
**Paired pulse facilitation in D1-MSNs is converted into paired-pulse depression by SNAP. (A)** A D1-MSN displayed typical electrophysiological properties when injected with current steps (20 pA increments). Rheobase current was 220 pA. **(B)** Neurobiotin staining of the neuron in A revealed dense dendritic arborizations and dendritic spines. Scale bar = 50 μm. **(C)** In a D1-MSNs, paired-pulse facilitation (at 50 ms interval) was observed in control solution (which contained GABA_A_ and GABA_B_ receptor antagonists), but was converted into paired-pulse depression by SNAP, that reduced the amplitude of the second evoked EPSP. These effects were reversed after SNAP washout. Each trace in this and subsequent figures showing paired pulse responses is the average of at least 50 consecutive responses. **(D)** Effects of SNAP on the first evoked response and the paired-pulse ratio of D1-MSNs that displayed paired-pulse facilitation in control solution. In this and in the following figures, asterisks indicate that the effects of certain treatment were statistical significant (*p < 0.05 and ***p < 0.001) in every cell tested. **(E)** In another D1-MSN, paired-pulse depression of corticostriatal responses was present in control solution. In this case, application of SNAP failed to significantly affect these responses. **(F)** Effects of SNAP on the first evoked response and the paired-pulse ratio of D1-MSNs that displayed paired-pulse depression in control solution.

D1 MSNs displayed distinctive anatomical features and membrane properties (including inward rectification and delayed firing; Figure 1A), as previously described (Gertler et al., 2008).

The average resting membrane potential in these neurons (*n* = 48, of which 30 from Drd1a-tdTomato mice and 18 from Drd2-eGFP mice) was −88 ± 6 mV; rheobase current was 238 ± 51 pA and input resistance at rest was 72 ± 29 MΩ. Figure 1B shows an example of neurobiotin filling for a D1-MSN from a Drd1a-tdTomato mouse.

In order to preserve the physiological interactions between synaptic inputs and dendritic and somatic voltage-dependent conductances, we studied short-term plasticity of cortical inputs to MSNs under current-lamp conditions without injection of current.

Paired-pulse stimulation of corticostriatal afferents was delivered as described in the “Materials and Methods” Section. In control solution, paired-pulse depression (average PPR < 1) was the prevalent type of response for D1-MSNs. Paired-pulse facilitation (average PPR > 1) was observed in 16/48 D1-MSNs (33%), while paired-pulse depression was observed in 32/48 D1-MSNs (67%). Examples of facilitating and depressing responses are shown in Figures 1C,E respectively.

We studied the effects of SNAP on evoked glutamatergic responses in subpopulations of D1-MSNs that expressed either paired-pulse depression or paired-pulse facilitation in control solution.

In 8/8 D1-MSNs in which paired-pulse facilitation was observed in control solution (below referred to as “facilitating MSNs”), bath application of the nitric oxide donor SNAP (100 μM) significantly (*p* < 0.001) decreased the amplitude of the second evoked response, but did not affect the first evoked response, as shown in the example of Figure 1C. As a result, the paired-pulse ratio was significantly (*p* < 0.001) reduced in the presence of SNAP, so that paired-pulse facilitation was replaced by paired-pulse depression, in all cases. Results of individual experiments on facilitating D1-MSNs are plotted in Figure 1D.

Conversely, in 6/6 D1-MSNs in which paired-pulse depression was observed in control solution, SNAP did not affect significantly the amplitude of either the first or the second evoked response, as shown in the example of Figure 1E. Accordingly, the paired-pulse ratio was not significantly different in the presence of SNAP in any cell. Results of individual experiments on depressing D1-MSNs are plotted in Figure 1F.

In voltage-clamp experiments, SNAP did not significantly affect the amplitude or frequency of miniature EPSCs in D1-MSNs (*n* = 6; in 4 of these 6 D1-MSNs, SNAP was re-applied after complete washout in a previous current clamp experiment). D1-MSN membrane potential/holding current and input resistance were also not significantly affected by SNAP in any of the neurons tested in current-clamp or voltage-clamp conditions (*n* = 16).

### Effects of SNAP on D2-MSNs

**Figure 2:**
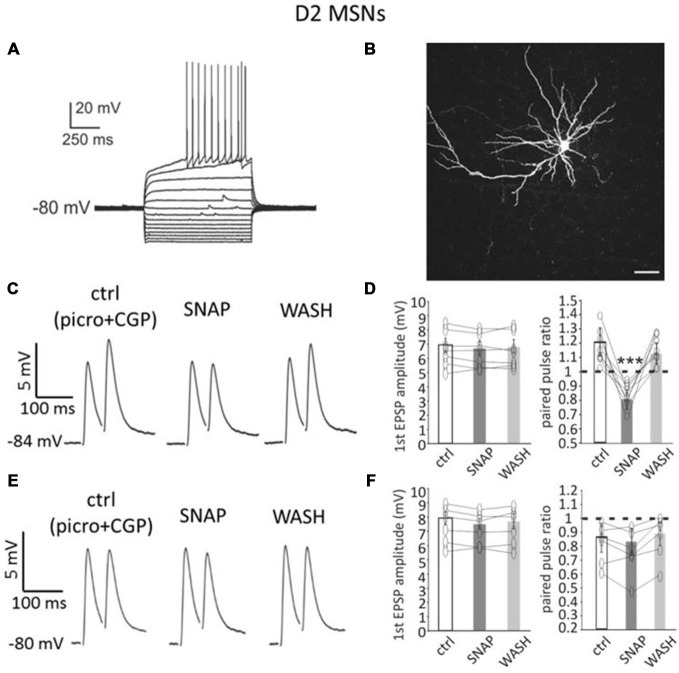
**SNAP converted paired-pulse facilitation into paired-pulse depression in D2-MSNs. (A)** Responses of a D2-MSN to current injections (20 pA increments). Rheobase current was 80 pA. **(B)** Neurobiotin staining of the neuron in A revealed its dendritic arborization and spines. Scale bar = 50 μm. **(C)** In a D2-MSNs paired-pulse facilitation was present in control solution (which contained GABA_A_ and GABA_B_ receptor antagonists), but was reversibly converted into paired-pulse depression by SNAP, that reduced the amplitude of the second evoked EPSP. Each trace in this and other figures showing paired pulse responses is the average of 50 consecutive responses. **(D)** Effects of SNAP on the first evoked response and the paired-pulse ratio of D2-MSNs that displayed paired-pulse facilitation in control solution. **(E)** In another D2-MSN, paired-pulse depression of corticostriatal responses was present in control solution. In this case, application of SNAP failed to significantly affect these responses. **(F)** Effects of SNAP on the first evoked response and on paired-pulse ratio of D2-MSNs that displayed paired-pulse depression in control solution.

D2-MSNs also displayed distinctive anatomical and membrane properties similar to, but distinct from, those of D1-MSN (Figure 2A), as previously described (Gertler et al., 2008). The average resting membrane potential in these neurons (*n* = 38, of which 26 from Drd1a-tdTomato mice and 12 from Drd2-eGFP mice) was −81 ± 8 mV; rheobase current was 110 ± 46 pA and input resistance at rest was 115 ± 20 MΩ. An example of neurobiotin filling for a D2-MSN from a Drd1a-tdTomato mouse is presented in Figure 2B.

In D2-MSNs, paired-pulse facilitation was prevalent in control solution. Paired-pulse facilitation (average PPR > 1) was observed in 27/38 D2-MSNs (71%), while paired-pulse depression (average PPR < 1) was observed in 11/38 D2-MSNs (29%).

In 7/7 D2-MSNs in which paired-pulse facilitation was observed in control solution, SNAP application did not significantly affect the first evoked response, but decreased the amplitude of the second evoked response, as shown in the example of Figure 2C. As a consequence, in all facilitating D2-MSNs, the paired-pulse ratio was significantly (*p* < 0.001) reduced in the presence of SNAP and paired-pulse facilitation was replaced by paired-pulse depression. Results of individual experiments on D2-MSNs that displayed paired-pulse facilitation in control solution are shown in Figure 2D.

In contrast with these effects, in 6/6 D2-MSNs in which paired-pulse depression was observed in control solution, SNAP did not affect the amplitude of either the first or the second evoked response, as shown in the example of Figure 2E. Consequently, the paired-pulse ratio was not significantly different from control in the presence of SNAP.

Results of individual experiments for D2-MSNs that displayed paired-pulse depression in control solution are plotted in Figure 2F.

In voltage-clamp experiments, SNAP did not significantly affect the amplitude or frequency of miniature EPSCs in D2-MSNs (*n* = 6; in 5 of these 6 D2-MSNs, SNAP was re-applied after its complete washout in a previous current-clamp experiment). Furthermore, SNAP did not significantly affect D2-MSNs membrane potential/holding current or input resistance in any of the cells tested under current-clamp or voltage clamp conditions (*n* = 14), suggesting that the observed effects originated at the level of the presynaptic network.

### Effects of SNAP on Cholinergic Interneurons

Whole-cell recordings in rats have shown that NO donors excite cholinergic interneurons (Centonze et al., 2001). We replicated these experiments in mouse brain slices using whole-cell recordings as well as cell-attached recordings, a technique that minimizes disruption of the intracellular environment. Overall, data were pooled from 24 cholinergic interneurons, of which four from Drd1a-tdTomato mice and 20 from Drd2-eGFP mice. In both recording configurations (15 whole-cell recordings and 9 cell-attached recordings), SNAP strongly and reversibly increased cholinergic interneuron firing frequency. The interspike interval was significantly (*p* < 0.001) reduced in the presence of SNAP in all cases and these effects were reversed after SNAP washout.

**Figure 3:**
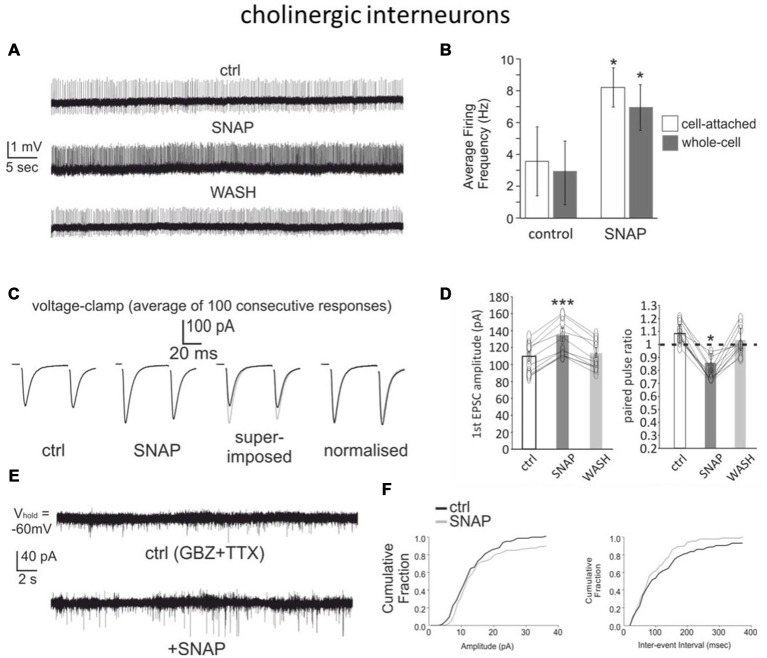
**SNAP excites cholinergic interneurons and facilitates their glutamatergic inputs. (A)** Bath application of SNAP caused a strong increase in spontaneous firing frequency in a cholinergic interneuron recorded in cell-attached configuration. **(B)** Average frequency of spontaneous spikes in cholinergic interneurons recorded in cell-attached or whole-cell configurations in control solution and in the presence of SNAP. A significant (*p* < 0.05) increase was observed in each interneuron tested. **(C)** SNAP increased the glutamatergic responses evoked in voltage-clamp by paired-pulse stimulation of corticostriatal fibers (each trace is the average of 100 consecutive responses). **(D)** Effects of SNAP on the amplitude of the first response and the paired-pulse ratio in cholinergic interneurons. **(E)** Effects of SNAP on miniature EPSCs in a cholinergic interneuron. **(F)** Average effects of SNAP on the cumulative probability of amplitude and inter-event interval distribution of spontaneous miniature EPSCs. The average amplitude and frequency of mini EPSCs were both significantly larger in the presence of SNAP.

An example of cell-attached experiments is shown in Figure 3A, while the overall effects are quantified in Figure 3B.

We also investigated whether SNAP affected glutamatergic responses evoked in cholinergic interneurons with a paired-pulse protocol (50 ms interval). Given the typical instability of cholinergic interneurons’ membrane potential (Blomeley and Bracci, 2005), these experiments were carried out under voltage-clamp conditions. Bath application of SNAP caused a significant increase in the amplitude of the first evoked responses in 12/12 cholinergic interneurons, while the second response was significantly increased in 7/12 cells and not significantly affected in the remaining five cells. The paired-pulse ratio was significantly (*p* < 0.05) reduced in the presence of SNAP in 10/12 cholinergic interneurons, suggesting that the facilitating effects of SNAP on the glutamatergic responses of cholinergic interneurons originated presynaptically. These effects are illustrated in Figures 3C,D. Consistent with this finding, SNAP significantly increased both the frequency and the amplitude of spontaneous miniature glutamatergic EPSCs in 6/6 cholinergic interneurons, as illustrated in Figures 3E,F. Overall, these data show that a nitric oxide donor strongly increased the firing of cholinergic interneurons and facilitated their cortical glutamatergic inputs.

### Effects of Cholinergic Antagonists on MSN Paired Pulse Facilitation

These observations suggested that cholinergic interneurons may be involved in the effects of SNAP on the responses of the MSNs exhibiting paired-pulse facilitation in control solution. To test this hypothesis, we studied whether the effects of SNAP on the glutamatergic inputs of facilitating MSNs were affected by antagonists of muscarinic or nicotinic ACh receptors.

**Figure 4:**
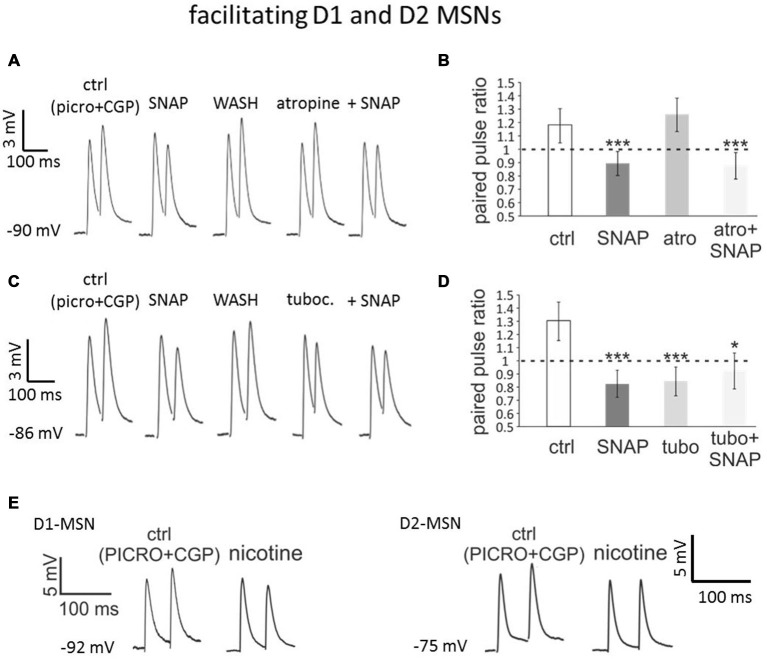
**Blocking nicotinic receptors mimics and occludes the effects of SNAP on paired-pulse facilitation in D1 and D2-MSNs. (A)** In a D2-MSN, paired-pulse facilitation was converted into paired-pulse depression by SNAP. After washout of the SNAP effects, application of atropine failed to affect the evoked responses. In the presence of atropine, SNAP had similar effects as when applied in control solution. Each trace is the average of 50 consecutive responses. **(B)** Average effects on paired-pulse ratio of SNAP, atropine and combined SNAP plus atropine application in a population of facilitating cells comprising four D1-MSNs and eight D2-MSNs. In each neuron tested, the paired-pulse ratios were significantly lower in SNAP and in atropine plus SNAP than in control. **(C)** In another D2-MSN, paired-pulse facilitation was converted into paired-pulse depression by SNAP. After washout of the SNAP effects, application of tubocurarine also converted paired-pulse facilitation into paired-pulse depression. When applied in the presence of tubocurarine, SNAP failed to affect the evoked responses. **(D)** Effects on paired-pulse ratio of SNAP, tubocurarine and SNAP plus tubocurarine in a population of facilitating MSNs comprising four D1-MSNs and eight D2-MSNs. In each neuron tested, the paired-pulse ratios was significantly lower in the presence of SNAP alone, tubocurarine alone or tubocurarine plus SNAP than in control solution. **(E)** Bath-application of nicotine, that induces profound desensitization of nicotinic receptors, converted paired-pulse facilitation into paired-pulse depression in a D1-MSNs (left) and a D2-MSN (right).

In a series of experiments on facilitating MSNs (*n* = 12, of which 3 D1-MSNs from Drd1a-tdTomato mice, 2 D2-MSNs from Drd1a-tdTomato mice, 1 D1-MSNs from Drd2-eGFP mice and 6 D2-MSNs from Drd2-eGFP mice), we compared the effects of SNAP in control solution and in the presence of the non-specific muscarinic receptor antagonist atropine (25 μM). In these experiments, SNAP was initially applied in the absence of atropine, which caused a significant (*p* < 0.001) and reversible reduction of the paired-pulse ratio in each of 12 neurons tested without affecting the first evoked EPSP, as shown in the representative example of Figure 4A. After complete washout of SNAP effects, atropine was applied. Atropine *per se* had variable effects on the evoked EPSPs, but did not significantly affect the paired-pulse ratio in any of the cells tested, as illustrated in Figure 4A. Re-application of SNAP in the presence of atropine resulted again in a significant (*p* < 0.001) reduction of the paired-pulse ratio in all cells tested, without significant effects on the first evoked EPSP. The average effects of SNAP on the paired-pulse ratios of facilitating MSNs before and after atropine application are shown in Figure 4B. The effects of SNAP on the paired-pulse ratios were similar in control solution and in the presence of atropine. We concluded that the ability of SNAP to reduce paired-pulse ratio in facilitating MSNs did not depend on activation of muscarinic receptors.

In a second series of experiments on facilitating MSNs (*n* = 12 of which 3 D1-MSNs from Drd1a-tdTomato mice, 4 D2-MSNs from Drd1a-tdTomato mice, 1 D1-MSNs from Drd2-eGFP mice and 4 D2-MSNs from Drd2-eGFP mice), a similar protocol was used to study the effects of the nicotinic receptor antagonist tubocurarine (10 μM). In the absence of tubocurarine, SNAP did not affect the amplitude of the first evoked response, but decreased the second evoked response, so that paired-pulse depression replaced facilitation in all MSNs tested (*p* < 0.001 for the paired pulse ratios). SNAP was then washed-out and its effects on paired-pulse ratio were reversed. Subsequent application of tubocurarine did not significantly affect the amplitude of the first evoked EPSP but reduced the second evoked EPSP, so that the paired-pulse ratio was significantly (*p* < 0.001) reduced in each of the neurons tested. A second application of SNAP, this time in the presence of tubocurarine, failed to further affect the paired-pulse ratio in any of these cells. As a result, in the presence of SNAP and tubocurarine, the PPR was significantly (*p* < 0.05) smaller than in control solution. These results are illustrated in the representative example of Figure 4C and quantified in Figure 4D. Thus, a nicotinic antagonist mimicked and occluded the effects of SNAP on paired-pulse ratios of facilitating MSNs. We concluded that paired-pulse facilitation in MSNs required functional nicotinic receptors and that the effects of SNAP were exerted through a functional inactivation of these receptors.

To further test the involvement of nicotinic receptors, we carried out some experiments in which these receptors were desensitized by bath-application of 500 nM nicotine (Sullivan et al., 2008). In both D1-MSNs (*n* = 4) and D2-MSNs (*n* = 4) exhibiting paired-pulse facilitation in control solution, nicotine converted such a facilitation into paired-pulse depression, as shown in the representative examples of Figure 4E. In all cases, the paired-pulse ratio was significantly (*p* < 0.01) decreased in the presence of nicotine. These results confirmed that nicotinic receptors are required for the expression of paired-pulse facilitation in MSNs.

## Discussion

The main finding of this study is that paired-pulse facilitation of glutamatergic responses, which is observed in both D1-MSNs and D2-MSNs upon stimulation of the CC, requires activation of nicotinic receptors and is converted into paired-pulse depression by nitric oxide.

**Figure 5:**
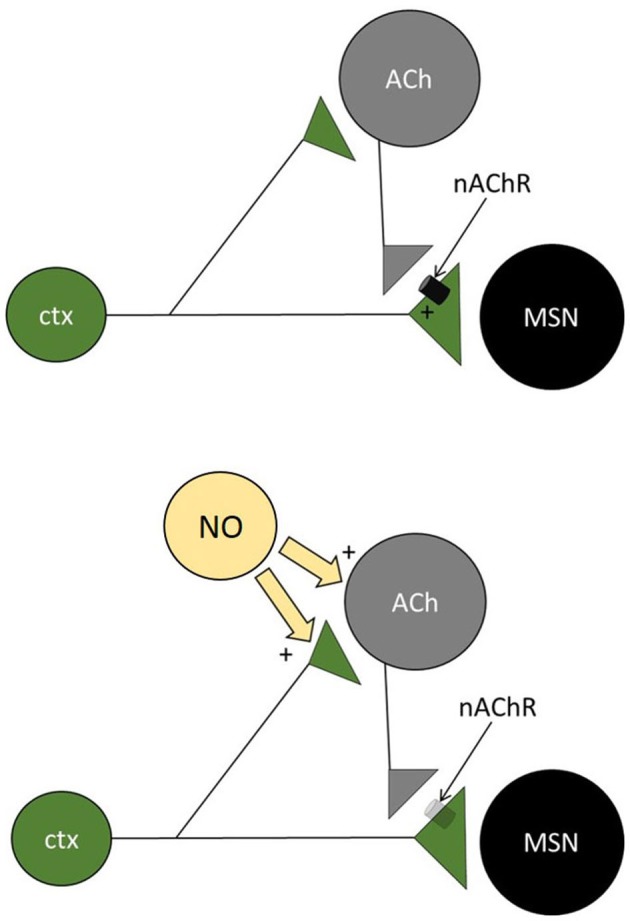
**Microcircuit underlying short term plasticity of MSN responses.** Diagram of the microcircuit proposed to be involved in the short-term plasticity of corticostriatal inputs to facilitating MSNs. Cortical inputs impinge on MSNs and on cholinergic interneurons (CI). In the absence of nitric oxide (top), ACh is released after the first stimulus and activates nicotinic receptors on the cortical terminals impinging on MSNs. As a result, the second pulse causes a larger release of glutamate from these terminals (paired-pulse facilitation). When nitric oxide is released by nitrergic interneurons (bottom), cholinergic interneurons are excited, but presynaptic nicotinic receptors are no longer activated, possibly as a result of vesicle depletion or receptor desensitization. As a result, the second pulse releases less glutamate than the first (paired-pulse depression).

We propose that paired-pulse facilitating responses in MSNs resulted from a microcircuit in which cholinergic interneurons produce a form of feed-forward facilitation (Figure 5). In this scheme, the first stimulus elicits supra-threshold EPSPs in local cholinergic interneurons, causing a synchronized action potential in these cells and subsequent activation of presynaptic nicotinic receptors on corticostriatal terminals impinging on MSNs. This activation then facilitates the response of MSNs to the second stimulus. In the presence of nitric oxide (released by LTS interneurons, that express nitric oxide synthase), cholinergic interneurons are excited but their ability to boost subsequent glutamate release in response to cortical inputs is lost (see below for possible explanations of this seemingly paradoxical phenomenon). The notion that evoked EPSPs that are subthreshold in MSNs can drive firing in cholinergic interneurons is consistent with the observation that these interneurons’ membrane potential is much closer to threshold than that of MSNs (Bennett et al., 2000). The finding that the miniature spontaneous glutamatergic events of MSNs were not affected by SNAP is consistent with the observation that the first evoked EPSP was not affected and supports the idea that a large, synchronized activation of cholinergic interneurons (like that achieved by electrical stimulation of afferent fibers) is required to cause feed-forward facilitation.

Under our experimental conditions, stimulation of the CC resulted in either paired-pulse depression or paired-pulse facilitation in subpopulations of both D1-MSNs and D2-MSNs, although depression was prevalent in D1-MSNs and facilitation was prevalent in D2-MSNs. In both MSN types, paired-pulse facilitation was converted into paired-pulse depression by SNAP or by tubocurarine, while paired-pulse depression was not affected by these agents. This suggests that similar intrastriatal circuits modulate the glutamate inputs on both direct and indirect pathway MSNs. Previous studies (using voltage-clamp techniques) had found that paired-pulse facilitation is expressed by corticostriatal fibers, while paired-pulse depression is typical of thalamostriatal fibers (Ding et al., 2008; Ellender et al., 2011). It is possible that, in our experiments, stimulation of the CC caused activation of a mixture of corticostriatal and thalamostriatal fibers. It is also possible that, under our current-clamp recording conditions, active dendritic conductances were activated by glutamatergic synaptic inputs and that this mechanism contributed to the observed paired-pulse ratios. Further experiments in which recording mode is switched from voltage- to current-clamp will be required to clarify if this is the case.

It is possible that a nitric oxide tone is present in the striatum in brain slices, as LTSIs are spontaneously active (Partridge et al., 2009; Ibáñez-Sandoval et al., 2011; Beatty et al., 2012). These interneurons may be also have been further excited by our stimulation protocols. However, nitric oxide release is believed to require prolonged depolarizations, and in some cases NMDA receptor activation (Garthwaite, 2008). The observed effects of SNAP on cholinergic interneurons and on glutamatergic transmission suggest that endogenous nitric oxide, if present, was not enough to cause saturation of the intracellular nitric oxide receptors responsible for the modulation observed here.

The engagement of cholinergic interneurons by cortical afferents in experiments in which paired-pulse facilitation was observed in MSNs is consistent with the observation that these neurons receive strong excitatory inputs from both the cortex and the thalamus (Doig et al., 2014). The lack of effects of nicotinic receptor antagonists in the experiments in which paired-pulse depression was observed in MSNs could be due to the fact that these neurons lacked the facilitatory presynaptic circuit in first place, possibly because the relevant afferent fibers were lost in brain slices or were not engaged by our stimulation. It is also possible that, as mentioned above, in these neurons the electrical stimulation activated predominantly thalamic inputs and that the feed-forward facilitation selectively controls corticostriatal fibers.

We confirmed in mice the observation originally made in rats that cholinergic interneurons are strongly excited by nitric oxide (Centonze et al., 2001). We also extended this observation by showing that both spontaneous and evoked glutamate inputs to cholinergic interneurons are potentiated by nitric oxide through a presynaptic mechanism. The observation that, in the presence of SNAP, paired-pulse facilitation was lost is apparently paradoxical. A nitric oxide donor had effects identical to those of a nicotinic receptor antagonist, despite having a strong excitatory influence on cholinergic interneurons, which are the main source of acetylcholine in the striatum. However, at least two plausible explanations can be formulated. In the presence of nitric oxide, the increased firing frequency of cholinergic interneurons may result in a dramatic depletion of ACh release form the terminals (synaptic fatigue). This scenario is strongly supported by recent results showing that nicotinic EPSPs recorded from striatal neurogliaform interneurons undergo profound but incomplete depression (~70%) when the presynaptic cholinergic interneurons are activated at 3.33 Hz (English et al., 2012). In our experiments, average firing frequency of cholinergic interneurons was around 3–4 Hz in control but doubled in the presence of SNAP. This increase in frequency could have increased synaptic depression to a point where spike-released ACh was no longer capable of significant activation of nicotinic receptors. Alternatively, nicotinic receptors may have become desensitized by persistently elevated concentrations of ACh. Desensitization of nicotinic receptors is well characterized and nicotine itself has been used as a nicotinic receptor *antagonist* in *in vitro* studies (Sullivan et al., 2008). The observation that bath-application of nicotine, known to cause profound desensitization of nicotinic receptors, affected paired-pulse responses in a manner similar to SNAP is consistent with the possible involvement of desensitization in the effects of the nitric oxide donor.

Regardless of the underlying cellular mechanism, these results demonstrate a novel role for both cholinergic and nitrergic neurons in the striatal network. Cholinergic interneurons appear to mediate feed-forward facilitation of excitatory inputs to MSNs, but this microcircuit can be switched off by nitric oxide, which is expected to be released by prolonged excitation of neurons expressing nitric oxide synthase (Garthwaite, 2008), that in the striatum correspond to LTSIs (Ibáñez-Sandoval et al., 2011). This nitrergic action would prevent facilitation of repetitive cortical inputs to MSNs, and may induce a general inactivation of nicotinic mechanisms in the local network.

The present results also raise the possibility that other neurotransmitters known to increase cholinergic interneuron firing, including serotonin (Blomeley and Bracci, 2005), may have a paradoxical antagonistic action on the activity of nicotinic (and possibly muscarinic) receptors. Further studies will be required to test this hypothesis.

From a functional point of view, our findings suggest that, in the absence of significant nitric oxide release, an initial volley of cortical inputs would cause facilitation of subsequent cortical inputs to MSNs. This process would be in functional competition with the feed-forward inhibition mediated by fast spiking GABAergic interneurons (Tepper et al., 2008; Szydlowski et al., 2013) and also with the presynaptic inhibition of cortical inputs that is mediated by cholinergic interneurons through activation of muscarinic receptors (Pakhotin and Bracci, 2007; Ding et al., 2010). Further studies will be required to reveal whether these different feedforward microcircuits impinge on the same MSNs and how they interact with feedback inhibitory circuits (Tepper et al., 2008) to shape the striatal outputs.

## Conflict of Interest Statement

The authors declare that the research was conducted in the absence of any commercial or financial relationships that could be construed as a potential conflict of interest.
